# Pathways to social inequality

**DOI:** 10.1017/ehs.2021.32

**Published:** 2021-07-08

**Authors:** Hannah J. Haynie, Patrick H. Kavanagh, Fiona M. Jordan, Carol R Ember, Russell D. Gray, Simon J. Greenhill, Kathryn R. Kirby, Geoff Kushnick, Bobbi S. Low, Ty Tuff, Bruno Vilela, Carlos A. Botero, Michael C. Gavin

**Affiliations:** 1 Department of Linguistics, University of Colorado, Boulder, CO, USA; 2 Department of Human Dimensions of Natural Resources, Colorado State University, Fort Collins, CO, USA; 3 Department of Anthropology and Archaeology, University of Bristol, Bristol, UK; 4 Human Relations Area Files, Yale University, New Haven, CT, USA; 5 Department of Linguistic and Cultural Evolution, Max Planck Institute for The Science of Human History, Jena, Germany; 6 ARC Centre of Excellence for the Dynamics of Language, Australian National University, Canberra, ACT, Australia; 7 Department of Ecology and Evolutionary Biology, University of Toronto, Toronto, ON, Canada; 8 School of Archaeology and Anthropology, Australian National University, Canberra, ACT, Australia; 9 School for Environment and Sustainability, University of Michigan, Ann Arbor, MI, USA; 10 Department of Biology, McGill University, Montreal, QC, Canada; 11 Institute of Biology, Universidade Federal da Bahia, Salvador, BA, Brazil; 12 Department of Biology, Washington University in St. Louis, St. Louis, MO, USA

**Keywords:** Social inequality, environmental conditions, resource intensification, wealth transmission, structural equation modelling

## Abstract

Social inequality is ubiquitous in contemporary human societies, and has deleterious social and ecological impacts. However, the factors that shape the emergence and maintenance of inequality remain widely debated. Here we conduct a global analysis of pathways to inequality by comparing 408 non-industrial societies in the anthropological record (described largely between 1860 and 1960) that vary in degree of inequality. We apply structural equation modelling to open-access environmental and ethnographic data and explore two alternative models varying in the links among factors proposed by prior literature, including environmental conditions, resource intensification, wealth transmission, population size and a well-documented form of inequality: social class hierarchies. We found support for a model in which the probability of social class hierarchies is associated directly with increases in population size, the propensity to use intensive agriculture and domesticated large mammals, unigeniture inheritance of real property and hereditary political succession. We suggest that influence of environmental variables on inequality is mediated by measures of resource intensification, which, in turn, may influence inequality directly or indirectly via effects on wealth transmission variables. Overall, we conclude that in our analysis a complex network of effects are associated with social class hierarchies.

## Graphical abstract

**Figure:**
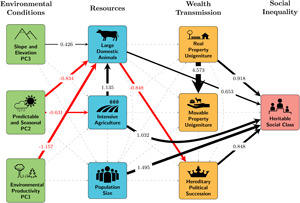

**Social media summary:** Web of environmental, resource intensification, wealth transmission and population size effects shape social inequality

## Introduction

Social and economic inequality are ubiquitous in contemporary human societies, a trend that has been linked to a number of detrimental consequences for the environment, the stability of political and economic systems and the well-being of individuals (Cushing, Morello-Frosch, Wander, & Pastor, 2015; Hurst, Fitz Gibbon, & Nurse, 2017; Karl, 2000). This inequality has been formalised and reinforced by cultural institutions like social class hierarchies and caste systems. However, human social organisation is commonly characterised as having consisted of essentially egalitarian, small-scale societies for the majority of human history (Bowles, Smith, & Borgerhoff Mulder, 2010; Flannery & Marcus, 2012; Hayden, 2001). While both external and intentional levelling mechanisms may have contributed to the pervasiveness of egalitarian, small-scale social organisation earlier in human history, in contrast to the strict social hierarchies common in other primate species (Boehm et al., 1993; Cashdan, 1980), a different set of mechanisms has been proposed to explain the emergence of social inequality and its widespread occurrence.

An extensive literature has focused on the reasons for the rise of inequality around the dawn of the Holocene (Bowles et al., 2010; Flannery & Marcus, 2012; Mattison, Smith, Shenk, & Cochrane, 2016; Sterelny, 2013). Somewhat less attention has been paid to whether mechanisms associated with the *de novo* origin of human social inequality might also shape the subsequent development and persistence of inequality in more recent societies. Here we examine if factors hypothesised to have shaped the early emergence of inequality have either direct or indirect influences on patterns of inequality in recently documented societies (see Figure 2). We focus on four sets of factors proposed by prior literature: environmental conditions, intensification in resource management, wealth transmission patterns and population size.

### The role of environmental conditions

The timing of the earliest evidence of human social inequality has been linked to patterns of climate change, and specifically a decline in climate variability around 12,000 years ago (Cohen, 1977). Some researchers have hypothesised that the shift to more stable and productive environmental conditions at the onset of the Holocene would have changed the relationships between humans and natural resources, and in the process reduced the need for risk mitigation strategies that previously levelled social hierarchies (Bowles & Choi, 2013; Kennett & Winterhalder, 2006; Richerson, Boyd, & Bettinger, 2001). This would imply that the mechanistic link between environmental conditions and inequality is mediated, at least in part, by subsistence strategies. However, the degree to which more productive and stable environmental conditions in the early Holocene contributed to shifts in subsistence, including the emergence of agriculture, is still widely debated (e.g. Kavanagh et al., 2018; Richerson et al., 2001). Research has shown a relationship between spatial patterns in environmental conditions and dominant subsistence strategies in recent human societies (Gavin et al., 2018). As we detail below, we might expect that intensification of subsistence activities would have consequences for the availability of resources, which can influence the distribution and accumulation of wealth, as well as the development of cultural institutions that reinforce inequality. In the current study we assess links between environmental conditions and inequality that are mediated via resource intensification and wealth transmission mechanisms.

### The role of intensification in resource management

Economic defensibility theory has been postulated to play a key role in the emergence of inequality in early Holocene small-scale societies. This principle of resource management entails a comparison between the costs of defending a resource patch through actions such as monitoring and preventing intruders, and the resulting benefits. Dense, predictable resources are more defensible, as they are associated with relatively small areas to defend, they are easy to locate and monitor, and the reliable and abundant resources they produce counter-balance the cost of defence. In early Holocene human groups, the scales may have tipped towards the adoption of these resource defence strategies when increasingly stable environmental conditions led to highly reliable or concentrated resource patches (Mattison et al., 2016). Over the subsequent millennia of cultural evolution, human innovations such as the domestication of livestock, the cultivation of agricultural resources and the intensification of production have further enhanced the density and predictability of resources, and thus their defensibility, in some societies (Smith et al., 2010). If the potential for resource intensification varies over space, it may serve as a critical driver of patterns of inequality, as some groups will have access to denser and more predictable resources than others. The domestication of plants and animals has been described as insufficient to spur the development of inequality (Smith et al., 2010), and evidence for inequality that predates agriculture suggests that it is also not a necessary condition for the emergence of inequality (Gurven et al., 2010; Hayden, 2001). Yet empirical work has found evidence that agriculture – particularly intensive agriculture – and the keeping of large domesticated animals is associated with the wealth distribution and transmission practices that shape social hierarchies (Borgerhoff Mulder et al., 2009; Kohler et al., 2017; Price, 1995; Shenk et al., 2010). We explore the direct links between resource intensification and social inequality, as well as the degree to which the effects of resource intensification are mediated by wealth transmission norms.

### The role of wealth transmission patterns

Intensification of subsistence strategies has also been associated with heritable forms of material wealth, including land and livestock, which have also been linked to higher levels of social inequality (Borgerhoff Mulder & Beheim, 2011). Intergenerational transmission of wealth allows unequal distributions of resources in a society to accumulate and persist over time, and is widely believed to play a role in social inequality in both ancient and contemporary societies (Borgerhoff Mulder et al., 2009; Bowles et al., 2010; Shennan, 2011; Sterelny, 2013). Wealth includes material assets like land and tangible property, as well as social wealth (e.g. support networks, power) and embodied wealth (e.g. physical health, knowledge) (Borgerhoff Mulder et al., 2009; Shenk et al., 2010). Material wealth is hypothesised to be particularly closely linked to inequality (Gurven et al., 2010; Shennan, 2011; Smith et al., 2010), especially in agricultural or pastoral societies (Borgerhoff Mulder et al., 2009; Smith et al., 2010). Inheritance of finite resources, like land, may also contribute to the asymmetries in resource distribution, and thus may be particularly important to the genesis of inequality (Smith et al., 2010). Although unequal wealth in the form of property rights may itself have emerged before agriculture (Bowles & Choi, 2013), the impacts of wealth transmission on social mobility may also play an important role in linking subsistence activities to institutionalisation of inequality. We test the hypothesis that the presence of norms favouring the hereditary transmission of material or social wealth will be shaped by environmental conditions and resource intensification, and will in turn influence the presence of institutionalised forms of social inequality.

### The role of population size

Another set of theories ascribes the rise of inequality to pressures associated with growing populations and the organisation of large-scale societies (Cohen, 1977; Johnson, 1982; Powers & Lehman, 2014; Turchin & Gavrilets, 2009). The scalar stress theory (Johnson, 1982), for example, associates the development of hierarchical organisation, including social status hierarchies, with the organisational pressures created by larger populations. Under such a theory we might expect population size to mediate impacts of agriculture or to serve as an independent driver of inequality. Population size may also impact economic defensibility in complex ways through its effects on within-group coordination and between-group competition (Chabot-Hanowell & Smith, 2013). For example, larger groups typically require more resources, and therefore they will benefit even more from resource intensification strategies. We incorporate population size into our model and assess both its direct and indirect effects on inequality; this allows us to examine whether resources and population work independently or in concert to impact wealth and social hierarchy, and whether one or the other of these is a more important driver of institutionalised inequality.

### Institutionalisation of inequality

Although inequality can be operationalised in a number of ways, we focus on a measure of heritable social class – an outcome that represents relatively rigid and persistent institutionalisation of inequality and is measured using a binary variable expressing the presence or absence of a social class system that is transferable across generations (see Methods). To be sure, some level of social and economic inequality may have existed throughout human history merely owing to individual variation in economic success and differences in the resources controlled by different families or lineages (Hayden, 2001). Persistent, institutionalised inequality includes a number of structures of varying levels of formality that emerge in societies to create and maintain stratification. While inequality exists in many forms, at many scales, in many parts of society, here we focus on a particular observable institution which requires widespread acceptance of persistent social hierarchies, is unequivocally associated with social inequality and can be reliably coded as present or absent across a wide range of societies (see Methods). We acknowledge that inequality takes many forms in human society and that alternative characterisations of inequality (e.g. Gini coefficients) are important for understanding other dimensions of inequality.

**Figure 1.**
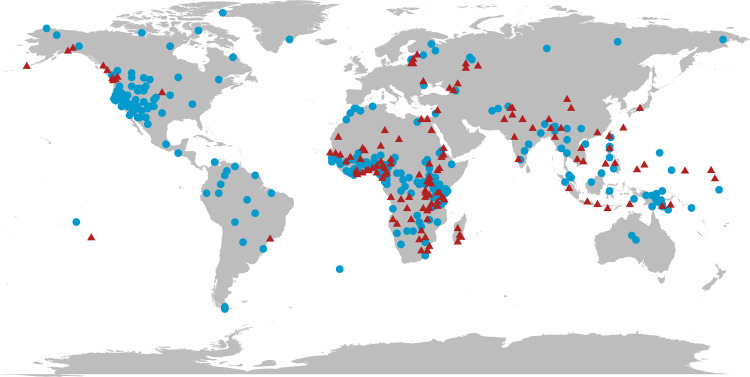
Societies included in the study (*n* = 408). Red triangles represent societies that are identified as having heritable social class systems. Blue dots represent societies with an absence of heritable social class.

**Figure 2.**
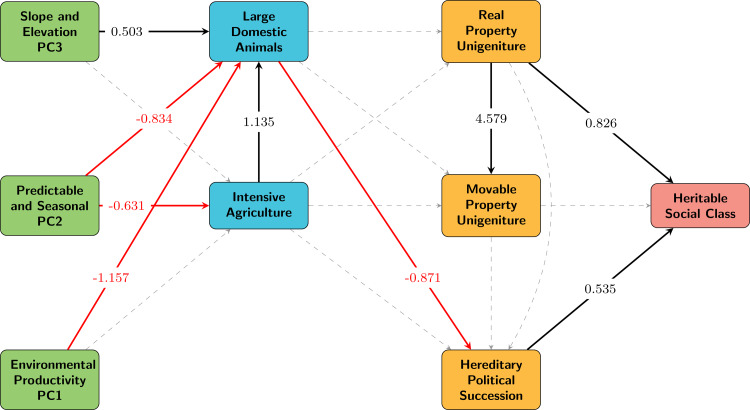
Path diagram representation of a strictly stepwise trajectory to social inequality (interpreted through variables derived from *D-PLACE*). Red arrows indicate negative relationships identified in PiecewiseSEM model. Black arrows represent positive relationships identified in PiecewiseSEM model. Dashed arrows represent paths not found to be significant (*P* < 0.05). Significant paths are labelled with standardised coefficients. Individual variables represented by boxes in the diagram can be interpreted as *increasing* for continuous variables and *present* for categorical variables. PC1 is associated with environmental productivity, PC2 with predictable and seasonal environments and PC3 with slope and elevation (see Methods for details on PCA-derived environmental variables). Fisher's *C* = 65.598, *P* = 0, conditional *R*^2^ for class = 0.30 (see Tables S3–S5 for full results); *n =* 408.

Using this framework, we examine the extent to which environmental conditions, resource intensification strategies, wealth transmission norms and population size have shaped institutionalised social inequality in recent human societies. We test a strict sequence, where the impacts of environmental conditions on inequality are mediated via effects on subsistence strategies and wealth transmission mechanisms in order, as implied by prior work on the early emergence of inequality (e.g. Mattison et al., 2016; Figure 2). We also examine the degree to which the impacts of environmental conditions, subsistence strategies and population size have direct effects on the presence of institutionalised inequality. These different relationships among potential drivers of inequality can be schematised as a path model (Figure 2). We investigate the details of each of the theoretical constructs in this framework using a large, global cross-cultural data sample from the *D-PLACE* database of places, languages, culture and environment (Kirby et al., 2016; Figure 1), and examine both incremental and direct effects of the associated variables on institutionalised inequality by applying a structural equation model (SEM) approach.

In testing the factors linked to inequality we use normative data at the society level to test predictions which are often derived from individual-level within-population phenomena. To the extent that we expect such phenomena to be detectable in population-level patterns, we see cross-cultural tests of behavioural ecology theory as an important source of evidence about these predictions (Kandler, Wilder, & Fortunato, 2017). By taking advantage of the large global sample of society-level data for a complex set of variables, we are able to simultaneously consider multiple facets of the generalised pathways and specific associations identified in prior literature (Borgerhoff Mulder et al., 2010; Mattison et al., 2016; Shenk et al., 2010).

## Methods

### Data

This study employs *Ethnographic Atlas* and environmental data for 408 societies available in the *D-PLACE* database (Colwell, 1974; Danielson & Gesch, 2011; Kirby et al., 2016; Lima-Ribeiro et al., 2015; Murdock, 1967; Running, Ramakrishna, Glassy, & Thornton, 1999; Figure 1) that are referenced to both specific times and places (see Supplementary Information for data descriptions and lists of societies and dates of sampling). The *Ethnographic Atlas* (available via *D-PLACE*) contains 1291 societies, for which 1106 have data on social class hierarchies. However, we only have complete data for all variables for a subset of these (*n =* 408). This sample of 408 societies is the maximal sample size for which all of the variables included in our study were available. While this sample is distributed around the globe, we did not control for cultural relatedness through sampling (as in, for example, the Standard Cross-Cultural Sample). Instead, we control for genealogical relationships explicitly in the design of our mixed effects path models. We use a random effect of language family in the SEM framework to control for well-established genealogical relationships between societies. The current lack of a reliable, global cultural phylogeny prevents us from using phylogenetic methods to control for shared histories.

The variables in the study serve as proxies for the more abstract constructs that are central to the hypothesised sequential evolution of early Holocene social inequality, and represent these societies as they were observed at a single point during or near the early twentieth century (see Table S1 for list of societies including dates). These data largely result from coding of ethnographic sources, which limits to some extent the ways in which we can test evolutionary hypotheses. For example, the variables selected for this study reflect not only a translation of central theoretical constructs to observable society-level phenomena, but also the availability of data describing those phenomena in a large sample of societies around the world. While the data and model we use do not explicitly reconstruct historical states of cultures and their changes over time, the relationships we identify in these empirical data enable inferences about the processes involved in the rise and maintenance of institutionalised social inequality in contemporary human cultures.

We represent environmental conditions using three variables derived from a principal component analysis (PCA). Although work on early Holocene inequality has characterised the environment largely in terms of temporal trends towards stability immediately prior to that time, the environmental variables included in this analysis allow us to characterise the productivity and predictability of local environments, which are likely to impact the spatial variation we find in human economic activity and cultural norms across a relatively narrow slice of history. Raw data used in this study describe the mean, variance, and predictability of temperature, precipitation and net primary productivity (NPP) at each location, as well as measures of elevation and slope (see Supplementary Information). Because this set of environmental variables is known to be highly correlated, we reduced it to three composite variables through PCA to avoid multicollinearity in the downstream SEM analyses (see Table S2). PC1 describes a gradient including variables associated with environmental productivity. Higher values of PC1 are associated with predictable and invariably warmer temperatures, seasonal and high levels of precipitation and high levels of NPP. PC2 describes a gradient associated with increasingly predictable and seasonal environments, including NPP variance and precipitation predictability. Higher values for environmental productivity and stability are hypothesised to produce conditions that can enhance resource intensification. PC3 describes a gradient including increasing slope and elevation. Higher values of topographic complexity may increase the patchiness of resources and may thus also contribute to defensibility (see Discussion for details).

The observed link between subsistence and inequality has been explained as an association between intensive agriculture and property rights, and a resulting concentration of material wealth and political power in agricultural societies (Bowles & Choi, 2013; Ember, Ember, & Russett, 1997; Price, 1995; Shenk et al., 2010; Smith et al., 2010). The presence of large domesticated animals represents a similar pattern that has been identified for pastoralist societies (Borgerhoff Mulder et al., 2010), and also an association between plow agriculture, the maintenance of draught animals and differential distribution of material wealth (Kohler et al., 2017). We include both the presence of large domesticated animals (recorded as a binary variable expressing the presence or absence of animals larger than sheep and goats, recoded from Ethnographic Atlas variable 040) and the presence of intensive agriculture (a binary variable expressing the presence or absence of intensive agricultural practices, recoded from Ethnographic Atlas variable 028) in our study to represent subsistence activities that have been linked to inequality in prior empirical studies and are likely to impact the economics of resource defence. Because the data we employ do not distinguish between animals used as a food resource and animals used for labour, we also report results using a dataset that excludes societies that obtain a majority of their subsistence through pastoralism (see Supplementary Information; Figure S3).

We focus on resource intensification as a technological and economic link between environmental conditions and wealth accumulation. While prior research has portrayed this link more broadly as a function of economic defensibility, targetting this mechanism allows this analysis to ask a specific question about the human activities that may have resulted in institutionalised inequality in contemporary human societies. Our analysis represents wealth transmission primarily as it relates to material and social wealth. Material wealth transmission is characterised for the purposes of this study by variables representing the presence or absence of inheritance rules that bequeath real property (land) (measured by a binary variable expressing the presence of a land inheritance rule assigning real property to a single heir, recoded from Ethnographic Atlas variable 075) and movable property (a binary variable expressing the presence of an inheritance rule assigning movable property to a single heir recoded from Ethnographic Atlas variable 077), respectively, to a single heir (unigeniture). Social wealth transmission is characterised here by the presence or absence of hereditary political succession. Although we recognise the importance of embodied wealth in shaping cultures, data limitations prevent us from exploring that component of wealth transmission in this analysis.

Real property has been ascribed a particularly prominent role in differential wealth distribution owing to practical limits on its subdivision and productivity (Shennan, 2011; Smith et al., 2010). Movable property may be relatively indefensible; however, possession and inheritance of animals have been linked to inequality (Borgerhoff Mulder et al., 2010; Kohler et al., 2017). We include both real and movable property to investigate the roles of each in the generation of institutionalised inequality. We hypothesise that these two types of wealth may interact in different ways with intensive agriculture and with the keeping of large animals. Our characterisation of material property inheritance in terms of unigeniture, which signifies wealth passed down to an individual with particular characteristics (e.g. the oldest child), reflects an expectation expressed by many authors that the inherently unequal pattern of wealth transmission across generations that unigeniture represents is particularly likely to concentrate resources and power and thus lead to institutionalised inequality (see Grieco and Ziebarth, 2015 and references therein). However, we recognise that other forms of intergenerational transmission, which pass wealth on to multiple individuals or groups, may not concentrate wealth as acutely each generation but could still serve to amplify inequality over time. In turn, we also tested our model with intergenerational property transmission encoded simply as the presence or absence of any inheritance rules for real and movable property. In using a variable that describes hereditary political succession to represent social wealth inheritance, we consider political power to be a reflection of social influence, and its hereditary assignment to be a manifestation of the intergenerational transmission of social wealth.

Class was encoded as a binary (presence/absence) variable expressing the presence or absence of a social class system that is transferable across generations (recoded from Ethnographic Atlas variable 066), so that relationships between individual types of wealth transmission and the specific inequality outcomes may be investigated. We restricted the presence category to instances where the social stratification system may persist across generations, namely heritable social class. This response variable represents a small subset of the outcomes that can be considered to exemplify persistent, institutionalised, inequality. However, class has the advantages of being reliably identifiable as a persistent and institutionalised form of inequality, of being recoverable for a large, globally distributed sample of societies, and of representing a particularly rigid and entrenched mechanism for enforcing social hierarchies.

We encoded population as a continuous variable that estimates the number of individuals in each entire ethnic group. More information about the coding of all variables can be found in the Supplementary Information.

### Statistical analysis

We analysed the data described above for 408 societies in the R statistical computing environment, using the packages PiecewiseSEM and lme4 for structural equation modelling (Bates, Mächler, Bolker, & Walker, 2015; Lefcheck, 2015). Language family was included as a random effect to control for potential non-independence of data that may result from common cultural inheritance, following Botero et al. (2014). Because no widely accepted global phylogeny of languages or cultures currently exists, we are unable to implement phylogenetic path models, and instead use a less complex but more widely accepted method of controlling for historical relationships through the inclusion of well-established language family classifications as a random effect in a mixed model framework (Botero et al., 2014).

Our study compares two alternative models, which vary in the number of variables and paths included on a standardised structure that reflects the general sequence and directionality of causal links proposed in prior literature to explain the emergence of inequality. In each path model the direction of causality in relationships between variables is assumed to follow a trajectory from environmental conditions to resource intensification to wealth transmission, resulting finally in inequality. The two models vary only in the inclusion of population size and the presence of individual pathways.

The first alternative (Figure 2) restricts the paths in the model to a stepwise trajectory that includes only those direct effects that represent sequential links between constructs in the aforementioned order, with no additional direct paths. In this model, environmental conditions, represented by our derived PCA variables, have direct effects only on subsistence (large domesticated animals and intensive agriculture). Subsistence variables represent the use of resources and technologies to intensify subsistence, and these variables in turn have direct effects only on wealth transmission variables. The three wealth transmission variables have direct effects only on the social inequality variable (i.e. class). Any relationship between environmental or subsistence variables and inequality can be characterised in this model only by an indirect path through one or more wealth transmission variables.

**Figure 3.**
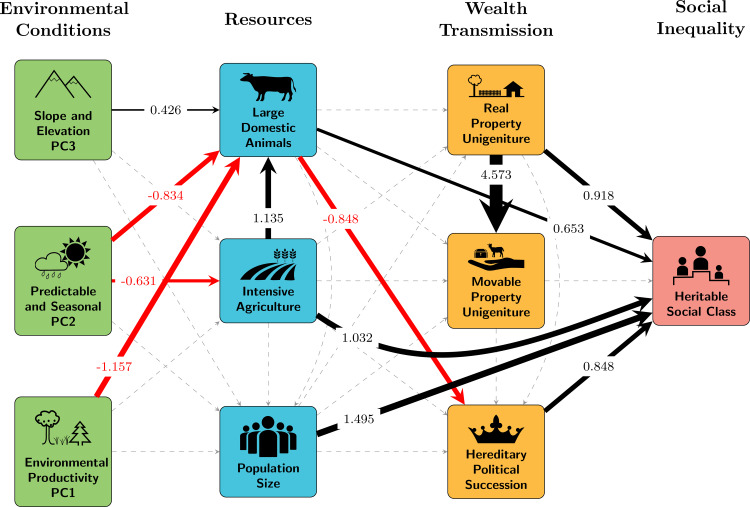
Path diagram representation of full model (interpreted through variables derived from *D-PLACE*). Black arrows represent positive relationships. Red arrows represent negative relationships. Dashed arrows represent paths not found to be significant (*P* < 0.05) Line weights indicate the estimated magnitude of effects and paths are labelled with standardised coefficients. Individual variables represented by boxes in the diagram can be interpreted as *increasing* for continuous variables and *present* for categorical variables. PC1 is associated with environmental productivity, PC2 with predictable and seasonal environments and PC3 with slope and elevation (see Methods for details on PCA-derived environmental variables). Conditional *R*^2^ for class = 0.45.

However, prior literature presents a more complex picture than the strictly stepwise schema is able to capture. For this reason we also consider a more elaborate model that adheres to the same assumptions about directionality and ordering of causal links, but includes a more complete set of direct and indirect paths between variables. In our second model (Figure 3), the directionality of all estimated paths still moves from environment to subsistence/population, then inheritance, and finally inequality. We added additional paths to the set used in the stepwise model to allow for the possibility of direct effects of predictors on variables farther to the right in the diagram when theoretical and/or empirical evidence existed for those direct pathways (see Discussion for comparison with prior findings). Specifically, direct paths extend from agricultural variables and population to social inequality variables. We did not add all of the paths called for by the directed separation tests from the stepwise model (see Results and Table S3), as many paths (e.g. direct paths from environmental variables to measures of intergenerational wealth transfer and to social inequality) did not have logical theoretical support or prior empirical evidence. As expected when we intentionally omit pathways owing to the absence of logical theoretical support, tests for global fit (direct separation tests and low *P*-value of Fishers C statistic) of our ‘full’ model may still indicate missing paths. In the second model population is treated as an additional potential predictor of wealth transmission and inequality variables, reflecting hypotheses that link inequality to demographic factors and the possibility that resource intensification and society scale have non-independent impacts on inequality outcomes. Direct paths also link both environmental variables and resource intensification variables to population.

## Results

Shipley's test for directional separation indicates that paths are missing from our first model that implemented a strictly stepwise progression of mechanisms on the pathway to inequality (Figure 2, Fisher's *C* = 65.598, d.f. = 28, *P* = 0; see Table S3 for directed separation results; Shipley, 2013).

We then added to the model those paths for which theoretical or empirical support exists (Figure 3; see Discussion for comparison with prior findings). We find five significant pathways leading to social inequality, as measured by heritable social class. The presence of both real property unigeniture (standardised path coefficient = 0.826, *P* < 0.05) and hereditary political succession (standardised path coefficient = 0.535, *P* < 0.01) is significantly associated with an increased probability of the presence of heritable social class within a society. Alternative coding of wealth transmission finds that the presence or absence of any form of real or moveable property inheritance is not significantly associated with the heritable social class (Figure S3).

Both measures that we used as proxies for resource intensification (large domesticated animals and intensive agriculture) are associated directly and indirectly with the presence of heritable social class. The presence of large domesticated animals was directly associated (standardised path coefficient = 0.653, *P* < 0.05) with an increased probability of heritable social class. However, indirectly the large domesticated animals were also associated with decreased probability of hereditary political succession, which is linked to a decreased probability in the presence of social class. When we focus more narrowly on non-pastoral animal husbandry, our results for the impacts of large domesticated mammals on class are qualitatively similar to those reported in Figure 3 (see Supplementary Information, Figure S3). Intensive agriculture directly links to social class (standardised path coefficient = 1.032, *P* < 0.001), and indirectly impacts the probability of social class via its positive association (standardised path coefficient = 1.135, *P* < 0.001) with large domesticated animals. Environmental variables influence inequality indirectly via significant links with both large domesticated animals and intensive agriculture (Figure 3). In addition, we find that larger populations (standardised path coefficient = 1.495, *P* < 0.05) are directly associated with a higher probability of the presence of heritable social class.

## Discussion

We find that a more complex model involving a web of both direct and indirect effects is better supported than a strict stepwise model (see results of directed separation tests in Table S3). In particular, we find not only that the effects of resource intensification are mediated by intergenerational wealth transmission mechanisms, as is suggested by a stepwise model, but also that resource intensification can directly shape inequality. As we explain below, the diverse pathways that our full model suggest often support prior findings or theory for individual relationships among the key variables we explored.

Our results indicate that environmental conditions impact inequality indirectly via activities that relate to resource intensification, namely intensive agriculture and keeping large domesticated animals. In our sample, environments with less seasonal and less predictable climates are more likely to be associated with these two variables, both of which represent subsistence activities that may increase resource defensibility. In addition, less productive environments are associated with increased probability of domesticating large mammals. These results contradict some prior theory on the early origins of inequality, which has been characterised primarily in terms of large-scale patterns in climate stability and environmental productivity that created better conditions for resource defence in the early Holocene (Bowles & Choi, 2013; Kennett & Winterhalder, 2006; Richerson et al., 2001). However, much debate still exists regarding the degree to which early forms of agriculture and animal husbandry, as mechanisms of resource defence, were associated with more or with less stable and productive environmental conditions (Kavanagh et al., 2018). Some argue that plant and animal domestication evolved in response to deteriorating and fluctuating environmental conditions (e.g. Bar-Yosef, 1998; Marshall & Hildbrand, 2002; Moore & Hillman, 1992). Of course, the factors that drove the origins of resource defence mechanisms may not be the same as those that have maintained these mechanisms in more recent times. One explanation for the links we find in the societies we study here may be that more intensive forms of agriculture, such as irrigation, can provide a means of ensuring more stable sources of subsistence when environmental conditions are less predictable (Porter and Marlowe, 2007).

In our sample, greater slope and elevation is associated with a greater propensity for domesticated large mammals. Others have emphasised pastoralists’ abilities to exploit areas of high topographic relief (Kardulias, 2015); however, we find the same link between topographic complexity and large domesticated animals even when we remove pastoralists from the sample (Figure S2). Some may argue that mountainous regions support lower densities of resources and less arable land (e.g. Acheson et al., 2015), which should make resource defence strategies less likely, contradicting our findings. Importantly though, environmental conditions are often patchy in mountains, where the relative abundance of different resources can shift rapidly over short distances and human groups may demonstrate niche partitioning by specialising on resources within a limited elevational range (Kavanagh et al., 2021). In addition, competition for patchy resources can be intense (Cashdan, 1983). Overall, the benefits of creating resource defence mechanisms may outweigh the costs in patchy mountainous regions, which may help explain why we find a greater probability of large animal domestication and prior work has also found more land ownership (another form of resource defence) in these regions (Kavanagh et al., 2021).

Our full model demonstrates multiple pathways by which resource intensification practices may impact inequality. While intensive subsistence activities might be expected to have indirect impacts on social inequality through positive associations with wealth transmission (Borgerhoff Mulder & Beheim, 2011), we also find evidence for strong direct impacts of intensive agriculture and domesticated large mammals on inequality (see Table 1). Although we know that inequality can arise even in the absence of agriculture (Gurven et al., 2010; Hayden, 2001; Smith et al., 2010), our results suggest that subsistence activities themselves are important contributors to the social and economic mechanisms out of which rigid inequality structures can arise, independent of wealth transmission patterns that consolidate resources and status for the few. This finding implies that social inequality may arise through multiple pathways, some of which are not dependent on differential accumulation of property and power through wealth transmission practices. Specialisation and division of labour in economies associated with intensive agriculture, for example, might create occupation-based stratification in wealth and prestige, regardless of how property or political power is transmitted across generations.

**Table 1.** Direct and indirect effect sizes for individual paths. Comparison of the direct and indirect effects in structural equation model in Figure 3 (standardised coefficients). Net indirect effects are calculated by multiplying coefficients along each indirect path that connects the predictor and the ultimate response and computing the sum of all indirect paths between predictor and response. Bold text indicates the effect of greater magnitude. See Methods for interpretation of PCA-derived environmental variables.

Response	Predictor	Direct	Indirect
Class	Population	**1**.**495**	
Class	Intensive agriculture	**1**.**032**	−0.075
Class	Real property unigeniture	**0**.**918**	
Class	Political succession	**0**.**848**	
Class	PC2 (Predictable and seasonal)		**−0**.**624**
Class	PC3 (Slope and elevation)		**0**.**105**
Class	Large domesticated animals	0.653	**−0**.**719**
Class	PC1 (Productivity)		**−0**.**028**

We also find indirect pathways through which resource intensification variables may be associated with inequality. Not surprisingly, given the use of large animals to support agricultural activities such as plowing, a greater probability of intensive agriculture increases the probability of large mammal domestication. Our model also predicts that societies that make use of large domesticated animals are less likely to have systems of hereditary political succession. Our supplementary analysis (Figure S2), which focuses more narrowly on non-pastoral animal husbandry, suggests that the direction of the relationship between large domesticated animals and inequality is not merely an artefact of how animal husbandry has been operationalised. If the use of large animals is positively linked to the development of inequality, as Kohler et al. have suggested for agriculturalists (Kohler et al., 2017) and Smith et al. have proposed for pastoralist societies (Smith et al., 2010), the negative association we find between large animals and transmission of social wealth trends in the opposite direction than we might expect for our sample. Our result also contrasts with prior research that demonstrates positive links between pastoralism, intergenerational transmission of multiple types of wealth and inequality (Borgerhoff Mulder et al., 2010). One possible cause for the inconsistencies in these results may lie in discrepancies in both the way in which inequality is measured across different studies (e.g. the studies cited above focused on material (relational and somatic) forms of inequality) and the levels at which the key variables are examined. For example, Borgerhoff Mulder et al. (2010) suggest that the economic inequalities measured at the individual level in their study may exist, and perhaps even legitimate, political equalities that exist at the group level in some pastoralist societies.

Our results also include strong evidence that wealth transmission norms, and particularly those that concentrate power and real property holdings, are positively associated with the institutionalisation of social inequality through class systems in which social status is inherited. The association of both real property unigeniture and hereditary political succession with heritable social class supports the hypothesis that many types of wealth contribute to the generation of inequality (Borgerhoff Mulder et al., 2009; Shenk et al., 2010). Of the wealth transmission variables included in this analysis, real property unigeniture (transmission of land holdings to a single heir) has the strongest net effect on heritable social class. In contrast, movable property unigeniture has no effects on social inequality. These findings support the notion that real property is central to the effect that material wealth transmission has on inequality (Gurven et al., 2010; Shennan, 2011; Smith et al., 2010). We note that unigeniture may be a particularly potent mechanism for concentrating wealth and encouraging formal systems of inequality (Grieco and Ziebarth, 2015). Our alternative model, in which intergenerational property transmission was encoded simply as the presence or absence of any inheritance rules for real and movable property, resulted in no effect of wealth transmission variables on inequality (see Supplementary Information). This result points to the possibility that unigeniture norms, which pass wealth on to individuals, may be a more important driver of inequality than other means of intergenerational property transmission in which wealth is passed on to multiple individuals or groups.

Alternatively, we might expect a population-driven explanation to be manifested in a trajectory like the one modelled here through effects of population size on institutionalised social inequality. We find that population size is strongly associated with the presence of social class hierarchies. This result supports the expectations of scalar stress theory (Johnson, 1982), which links the evolution of hierarchical organisation, including hierarchies related to social status, to stresses created by increasing populations. We might expect that resource intensification activities, such as intensive agriculture, would contribute to increases in population sizes, but our model shows no significant links in this regard. However, we also recognise that the direction of causation may tend in the opposite direction with increases in population size, placing pressures on resources that lead to the need for more resource intensification. Unfortunately, the structural equation approach used here does not allow us to test for feedback loops in which causality would flow in both directions.

The language family random effect does have a substantial impact on our full model (conditional *R*^2^ for class = 0.45 vs. marginal *R*^2^ for class = 0.31), implying that the societies that are more closely related may share the same presence (or absence) of social class hierarchies. One possible explanation for this effect is the impact of vertical cultural transmission, in which cultural traits such as class are passed across generations and therefore are more likely to be shared by closely related groups. Future work should seek to include finer-grained measures of relatedness among societies as more reliable global phylogenies become available to allow for such an approach.

The complex network of effects that we identify between environmental, subsistence, inheritance, population size and social inequality variables suggests that how we measure each of the core cultural constructs associated with a theoretical trajectory for the development of social inequality matters to our ability to investigate the processes that create and maintain the institutions that most rigidly support social hierarchies. *A priori* assumptions might have emphasised intensive agriculture as a means of increasing economic defensibility, and thus be a likely predictor of real property inheritance patterns and inequality in turn (Dye, 2010), which could have been tested in a simpler model. However, the inclusion of several variables to represent different components of the theory enables us to identify pathways that relate to a diverse set of hypotheses.

Our ability to measure the relevant characteristics of societies is limited in practice by the availability of cross-cultural data. With the data used in this analysis we may not be able to capture all of the complexity in the phenomena discussed in great detail in a vast collection of prior literature, or to capture additional phenomena that may contribute to inequality. The results presented here do not, in other words, rule out other possible pathways. This includes the possibility of reverse causality in the case of some of the relationships that we explored, which it is not possible to test with the structural equation modelling approach we used owing to limits on the inclusion of feedback loops among variables. In addition, many causal relationships have been proposed that may impact individual cultural traits and mediate relationships within this set of variables. For example, although our results do not support the potential importance of real property inheritance as a link from agriculture to social inequality, such a pathway might be detectable if specific other variables were included in the analysis. We also emphasise that deviations between prior theory and our results may be due in part to differences between factors that contribute to the emergence vs. the maintenance of cultural traits (Ross, Strimling, Ericksen, Lindenfors, & Mulder, 2016). Much of the theory and prior empirical work that we have drawn on here focuses on the emergence of social inequality in the early Holocene. However, we might expect that once a given trait, such as social class hierarchies, becomes established, the influence of the drivers that caused the emergence may become less relevant to its persistence.

Owing to limitations of data availability we are unable to address every possibility outlined in prior research. Because this approach focuses on a specific set of general, society-level phenomena, we urge caution in interpreting the results of this type of analysis; our model helps us understand influences on cultural traits but does not represent a singular, inevitable trajectory for the pointing to social inequality. Other facets of human culture and behavior are of vital importance in understanding individual systems of social class.

The methods that we implement here represent a rigorous way to explore the complex relationships between cultural phenomena that may interact both directly and indirectly, while also controlling for shared histories. The structural equation approach illustrated in this analysis makes it possible to examine in detail the empirical evidence for theories that have been proposed to explain the evolution of human culture.

## Supplementary material

For supplementary material accompanying this paper visit https://doi.org/10.1017/ehs.2021.32. click here to view supplementary material

## Research transparency and reproducibility

All data are available open-access online at https://d-place.org/. Variables names and codes from the D-PLACE database, as well as names of all societies included are available in Supplementary Information. Also see supplementary materials for data and R code.
