# Supplementary material for: Pathways to social inequality
Source: Evol Hum Sci. 2021 Jul 8;3:e35. doi: 10.1017/ehs.2021.32 (PMC10427274; doi:10.1017/ehs.2021.32)
Supplement: Supplementary file 1 [file S2513843X21000323sup.zip › S2513843X21000323sup002.pdf]

Supplementary Materials

Description of data

All data are available online at https://d-place.org/. The following variables from *D-PLACE* were included in the analysis:

Environmental variables

- Elevation (*15*)
- Slope (*15*)
- Annual mean precipitation (*16*)
- Annual precipitation variance (*16*)
- Precipitation predictability (*13*, *16*)
- Annual mean temperature (*16*)
- Annual temperature variance (*16*)
- Temperature predictability (*13*, *16*)
- Mean monthly NPP (*14*)
- Annual NPP variance (*14*)
- NPP predictability (*13*, *14*)

Demographic variables

- Population Size – a continuous variable from *EA202 Population size* (*12*) that encodes the total population of the ethnic group as a whole

Subsistence variables

- Intensive Agriculture – a binary variable expressing the presence or absence of intensive agricultural practices, recoded from *EA028 Agriculture intensity* (*12*).
  - 0: EA028 = 1 *Complete absence of agriculture* OR 2 *Casual agriculture* OR 3 *Extensive or shifting cultivation* OR 4 *Horticulture*
  - 1: EA028 = 5 *Intensive agriculture on permanent fields* OR 6 *Intensive cultivation largely dependent upon irrigation*
- Large Domesticated Animals – a binary variable expressing the presence or absence of animals larger than sheep and goats, recoded from *EA040 Domestic animals: type* (*12*).
  - 0: EA040 = 1 *Absence or near absence of domestic animals* OR 2 *Pigs only animal of consequence* OR 3 *Sheep and/or goats when larger animals absent*
  - 1: EA040 = 4 *Equine animals* OR 5 *Deer (e.g. reindeer)* OR 6 *Camels or related genera* OR 7 *Bovine animals*

Wealth transmission variables

- Real Property Unigeniture – a binary variable expressing the presence of a land inheritance rule assigning real property to a single heir, coded from *EA075 Inheritance distribution for real property* (*12*).

- 0: EA075 = 1 *Equal or relatively equal distribution of land among all members of the category* OR 9 *Absence of inheritance of real property*
- 1: EA075 = 2 *Exclusive or predominant inheritance by the member of the category adjudged best qualified* OR 3 *Ultimogeniture* OR 4 *Primogeniture*

- Movable Property Unigeniture – a binary variable expressing the presence of an inheritance rule assigning movable property to a single heir, coded from *EA077 Inheritance distribution for movable property* (*12*).
  - 0: EA077 = 1 *Equal or relatively equal distribution among all members of the category* OR 9 *Absence of inheritance of movable property*
  - 1: EA077 = 2 *Exclusive or predominant inheritance by the member of the category adjudged best qualified* OR 3 *Ultimogeniture* OR 4 *Primogeniture*
- Hereditary Political Succession – a binary variable expressing the presence of a hereditary rule for political succession to the office of local headman, recoded from *EA072 Political succession* (*12*).
  - 0: EA072 = 3 *Nonhereditary through appointment or higher authority* OR 4 *Nonhereditary on the basis of seniority* OR 5 *Nonhereditary through influence* OR 6 *Nonhereditary through election* or 7 *Nonhereditary through informal consensus* OR 9 *Absence of office resembling local headman*
  - 1: EA072 = 1 *Patrilineal heir* OR 2 *Matrilineal heir*

Inequality variables

- Heritable Social Class – a binary variable expressing the presence or absence of a social class system that is transferable across generations, recoded from *EA066 Class differentiation primary* (*12*).
  - 0: EA066 = 1 *Absence of significant class distinctions* OR 2 *Wealth distinctions*
  - 1: EA066 = 3 *Elite stratification* OR 4 *Dual stratification* OR 5 *Complex stratification*

Table S1: Societies in sample

EA ID	Society	Language_Family	Focal_Year
Aa1	!Kung	Kxa	1950
Aa2	Dorobo	Nilotic	1920
Aa3	Nama	Khoe-Kwadi	1840
Aa7	Naron	Khoe-Kwadi	1910
Ab1	Herero	Atlantic-Congo	1900
Ab10	Pondo	Atlantic-Congo	1936
Ab12	Zulu	Atlantic-Congo	1830
Ab13	Tswana	Atlantic-Congo	1880
Ab15	Pedi	Atlantic-Congo	1860
Ab19	Ambo	Atlantic-Congo	1910
Ab2	Swazi	Atlantic-Congo	1880
Ab21a	Kisama	Atlantic-Congo	1930
Ab3	Lozi	Atlantic-Congo	1890
Ab6	Venda	Atlantic-Congo	1900

Ab7	Nyaneka	Atlantic-Congo	1920
Ab8	Sotho	Atlantic-Congo	1860
Ab9	Ndebele	Atlantic-Congo	1870
Ac1	Ila	Atlantic-Congo	1920
Ac12	Chokwe	Atlantic-Congo	1920
Ac17	Suku	Atlantic-Congo	1910
Ac3	Bemba	Atlantic-Congo	1900
Ac30	Plateau Tonga	Atlantic-Congo	1940
Ac32	Kaonde	Atlantic-Congo	1920
Ac33	Lala	Atlantic-Congo	1940
Ac34	Luapula	Atlantic-Congo	1940
Ac37	Kunda	Atlantic-Congo	1950
Ac38	Nyanja	Atlantic-Congo	1910
Ac4	Kuba	Atlantic-Congo	1910
Ac8	Yombe	Atlantic-Congo	1930
Ad1	Bajuni	Atlantic-Congo	1950
Ad10	Shambaa	Atlantic-Congo	1910
Ad11	Bena	Atlantic-Congo	1930
Ad12	Gusii	Atlantic-Congo	1940
Ad14	Luguru	Atlantic-Congo	1930
Ad15	Iwa	Atlantic-Congo	1900
Ad17	Safwa	Atlantic-Congo	1920
Ad2	Nyoro	Atlantic-Congo	1950
Ad24	Gogo	Atlantic-Congo	1910
Ad26	Turu	Atlantic-Congo	1910
Ad27	Kwere	Atlantic-Congo	1880
Ad29	Hadimu	Atlantic-Congo	1930
Ad3	Chagga	Atlantic-Congo	1910
Ad32	Giriama	Atlantic-Congo	1900
Ad33	Pokomo	Atlantic-Congo	1900
Ad35	Meru	Atlantic-Congo	1940
Ad39	Sonjo	Atlantic-Congo	1950
Ad4	Kikuyu	Atlantic-Congo	1930
Ad46	Soga	Atlantic-Congo	1950
Ad47	Sumbwa	Atlantic-Congo	1900
Ad6	Nyakyusa	Atlantic-Congo	1930
Ad7	Ganda	Atlantic-Congo	1880
Ad9	Gisu	Atlantic-Congo	1900
Ae1	Amba	Atlantic-Congo	1950
Ae10	Banyaruanda	Atlantic-Congo	1910
Ae14	Ha	Atlantic-Congo	1950
Ae20	Ekonda	Atlantic-Congo	1940
Ae21	Kela	Bookkeeping	1920
Ae3	Fang	Atlantic-Congo	1910
Ae33	Ndaka Babali	Atlantic-Congo	1920
Ae4	Nkundo	Atlantic-Congo	1930
Ae42	Ngumba	Atlantic-Congo	1900
Ae44	Bubi	Atlantic-Congo	1920

Ae45	Kundu	Atlantic-Congo	1900
Ae47	Puku	Atlantic-Congo	1900
Ae5	Bamileke	Atlantic-Congo	1910
Ae50	Bamum	Atlantic-Congo	1920
Ae51	Banen	Atlantic-Congo	1940
Ae8	Rundi	Atlantic-Congo	1910
Af1	Fon	Atlantic-Congo	1890
Af10	Igbo	Atlantic-Congo	1930
Af11	Toma	Mande	1920
Af15	Kpelle	Mande	1920
Af16	Banyang	Atlantic-Congo	1910
Af19	Efik	Atlantic-Congo	1950
Af20	Ibibio	Atlantic-Congo	1910
Af24	Edo	Atlantic-Congo	1900
Af27	Eloyi	Atlantic-Congo	1950
Af36	Ewe	Atlantic-Congo	1900
Af39	Anyi	Atlantic-Congo	1900
Af40	Assini	Atlantic-Congo	1890
Af41	Attie	Atlantic-Congo	1890
Af42	Fante	Atlantic-Congo	1900
Af45	Avikam	Atlantic-Congo	1900
Af51	Gagu	Mande	1920
Af52	Guro	Mande	1920
Af6	Oyo Yoruba	Atlantic-Congo	1950
Af7	Bete	Atlantic-Congo	1950
Af8	Nupe	Atlantic-Congo	1930
Af9	Baule	Atlantic-Congo	1900
Ag1	Bambara	Mande	1920
Ag10	Konkomba	Atlantic-Congo	1940
Ag12	Nankanse	Atlantic-Congo	1910
Ag16	Banyun	Atlantic-Congo	1930
Ag18	Bijogo	Atlantic-Congo	1930
Ag19	Diola	Atlantic-Congo	1930
Ag21	Bassari	Atlantic-Congo	1910
Ag22	Serer	Atlantic-Congo	1920
Ag3	Dogon	Dogon	1930
Ag30	Bobo	Mande	1910
Ag31	Minianka	Atlantic-Congo	1910
Ag35	Kulango	Atlantic-Congo	1910
Ag38	Builsa	Atlantic-Congo	1910
Ag39	Dagara	Atlantic-Congo	1910
Ag4	Tallensi	Atlantic-Congo	1930
Ag40	Isala	Atlantic-Congo	1910
Ag42	Nunuma	Atlantic-Congo	1910
Ag47	Mossi (Ouagadougou)	Atlantic-Congo	1900
Ag48	Basari	Atlantic-Congo	1890
Ag49	Kabre	Atlantic-Congo	1930
Ag50	Moba	Atlantic-Congo	1910

Ag54	Yalunka	Mande	1910
Ag8	Coniagui	Atlantic-Congo	1910
Ag9	Malinke	Mande	1930
Ah1	Katab	Atlantic-Congo	1930
Ah13	Dakakari	Atlantic-Congo	1930
Ah15	Reshe	Atlantic-Congo	1920
Ah16	Angas	Afro-Asiatic	1900
Ah19	Adara	Atlantic-Congo	1930
Ah2	Jukun	Atlantic-Congo	1920
Ah26	Ndoro	Atlantic-Congo	1920
Ah29	Daka	Atlantic-Congo	1920
Ah3	Tiv	Atlantic-Congo	1920
Ah30	Longuda	Atlantic-Congo	1920
Ah31	Mumuye	Atlantic-Congo	1910
Ah34	Bata	Afro-Asiatic	1920
Ah36	Gude	Afro-Asiatic	1920
Ah38	Kapsiki	Afro-Asiatic	1930
Ah39	Podokwo	Afro-Asiatic	1950
Ah5	Margi	Afro-Asiatic	1930
Ah7	Mafa	Afro-Asiatic	1940
Ah9	Anaguta	Atlantic-Congo	1920
Ai23	Bwaka	Atlantic-Congo	1920
Ai24	Mandja	Atlantic-Congo	1900
Ai26	Ngbandi	Atlantic-Congo	1920
Ai28	Popoi	Central Sudanic	1910
Ai29	Lendu	Central Sudanic	1920
Ai3	Azande	Atlantic-Congo	1920
Ai30	Lese	Central Sudanic	1920
Ai33	Madi	Central Sudanic	1940
Ai37	Koalib	Heiban	1940
Ai40	Moro	Heiban	1940
Ai6	Shilluk	Nilotic	1900
Ai7	Gbaya	Atlantic-Congo	1910
Aj15	Kuku	Nilotic	1900
Aj17	Alur	Nilotic	1890
Aj19	Didinga	Surmic	1920
Aj2	Maasi	Nilotic	1900
Aj23	Plains Suk	Nilotic	1950
Aj26	Hill Suk	Nilotic	1910
Aj28	Tatoga	Nilotic	1940
Aj29	Samburu	Nilotic	1950
Aj3	Nuer	Nilotic	1930
Aj4	Lango	Nilotic	1920
Aj5	Turkana	Nilotic	1920
Aj6	Luo	Nilotic	1940
Aj7	Nandi	Nilotic	1910
Aj8	Bari	Nilotic	1920
Aj9	Kipsigis	Nilotic	1920

Ca10	Esa	Afro-Asiatic	1880
Ca3	Tigrinya	Afro-Asiatic	1950
Ca32	Nara	Nara	1860
Ca33	Kunama	Kunama	1860
Ca38	Tigre	Afro-Asiatic	1860
Ca39	Jimma	Ta-Ne-Omotic	1930
Ca4	Iraqw	Afro-Asiatic	1950
Ca42	Shako	Dizoid	1950
Ca7	Amhara	Afro-Asiatic	1950
Cb19	Kanuri	Saharan	1870
Cb2	Wolof	Atlantic-Congo	1950
Cb23	Tukulor	Atlantic-Congo	1930
Cb25	Tazarawa	Afro-Asiatic	1930
Cb26	Zazzagawa Hausa	Afro-Asiatic	1950
Cb3	Songhai	Songhay	1940
Cb5	Buduma	Afro-Asiatic	1910
Cb8	Bororo Fulani	Atlantic-Congo	1920
Cc1	Regeibat	Afro-Asiatic	1950
Cc10	Asben	Afro-Asiatic	1900
Cc11	Azjer	Afro-Asiatic	1860
Cc13	Udalan Tuareg	Afro-Asiatic	1920
Cc2	Teda	Saharan	1950
Cc3	Siwans	Afro-Asiatic	1920
Cd10	Zekara	Afro-Asiatic	1900
Cd15	Jebala	Afro-Asiatic	1910
Cd18	Saadi	Afro-Asiatic	1920
Cd2	Egyptians	Afro-Asiatic	1950
Cd3	Riffians	Afro-Asiatic	1920
Cd4	Kabyle	Afro-Asiatic	1890
Cd5	Shluh	Afro-Asiatic	1920
Cd7	Beraber	Afro-Asiatic	1910
Cf3	Tristan da Cunha	Indo-European	1930
Cf4	Brazilians	Indo-European	1940
ch12	Bashkir	Turkic	1899
ch13	Besermyan	Uralic	1894
ch14	Chuvash	Turkic	1908
ch15	Erzya Mordvins	Uralic	1895
ch16	Estonians	Uralic	1908
ch17	Gagauz	Turkic	1894
ch18	Ingrians	Uralic	1927
ch19	Karelians	Uralic	1874
ch20	Kazan tatar	Turkic	1910
ch21	Latvians	Indo-European	1881
ch22	Lithuanian Karaim	Turkic	1895
ch23	Lithuanian Tatar	Turkic	1905
ch24	Livs	Uralic	1847
ch25	Moldovans	Indo-European	1900
ch26	Udmurt	Uralic	1890

ch27	Veps	Uralic	1900
ch28	Votes	Uralic	1900
Ch9	Lithuanians	Indo-European	1930
Ci1	Kalmyk	Mongolic	1920
Ci2	Khevsur	Kartvelian	1930
Ci4	Cherkess	Abkhaz-Adyge	1920
Ci6	Ossetians	Indo-European	1880
Cj7	Lebanese	Afro-Asiatic	1950
Cj8	Druze	Afro-Asiatic	1930
Ea10	Marri	Indo-European	1950
Ea13	Punjabi	Indo-European	1950
Ea2	Yusufzai	Indo-European	1950
Ea3	Hazara	Indo-European	1930
Ea4	Kohistani	Indo-European	1950
Ea5	Nuristani	Indo-European	1890
Ea7	Moghol	Mongolic	1950
Eb2	Tu	Mongolic	1920
Ec2	Yakut	Turkic	1900
Ec3	Chukchi	Chukotko-Kamchatkan	1900
Ec8	Ket	Yeniseian	1900
Ed1	Koreans	Koreanic	1950
Ed4	Miao	Hmong-Mien	1940
Ed5	Japanese	Japonic	1950
Ed6	Min Chinese	Sino-Tibetan	1920
Ed7	Okinawans	Japonic	1950
Ed8	Minchia	Sino-Tibetan	1930
Ed9	Li	Tai-Kadai	1930
Ee3	Lepcha	Sino-Tibetan	1930
Ee4	Tibetans	Sino-Tibetan	1920
Ef1	Santal	Austroasiatic	1940
Ef11	Uttar Pradesh	Indo-European	1945
Ef5	Bhil	Indo-European	1900
Ef7	Pahari	Indo-European	1950
Eg1	Chenchu	Dravidian	1940
Eg3	Madia	Dravidian	1938
Eg4	Toda	Dravidian	1900
Eg5	Kodavas	Dravidian	1930
Eh2	Merina	Austronesian	1900
Eh3	Tanala	Austronesian	1930
Eh7	Antandroy	Austronesian	1900
Eh9	Antaisaka	Austronesian	1930
Ei11	Aimol	Sino-Tibetan	1910
Ei14	Ao	Sino-Tibetan	1920
Ei19	Chin	Sino-Tibetan	1940
Ei2	Lhota	Sino-Tibetan	1920
Ei5	Kachin	Sino-Tibetan	1940
Ei6	Purum	Sino-Tibetan	1930

Ei7	Karen	Sino-Tibetan	1910
Ei8	Khasi	Austroasiatic	1900
Ej11	Cham	Austronesian	1950
Ej14	Senoi	Austroasiatic	1960
Ej3	Semang	Austroasiatic	1920
Ej4	Annamese	Austroasiatic	1950
Ej5	Khmer	Austroasiatic	1950
Ej7	Akha	Sino-Tibetan	1950
Ej9	Thai	Tai-Kadai	1940
Ia12	Sugbuanon	Austronesian	1950
Ia14	Tao	Austronesian	1930
Ia16	Kalinga	Austronesian	1910
Ia2	Sagada	Austronesian	1950
Ia3	Ifugao	Austronesian	1920
Ia4	Subanun	Austronesian	1950
Ia5	Hanunoo	Austronesian	1950
Ia6	Paiwan	Austronesian	1930
Ia7	Tagbanua	Austronesian	1950
Ia8	Bontok	Austronesian	1900
Ib1	Iban	Austronesian	1950
Ib2	Javanese	Austronesian	1950
Ib3	Balinese	Austronesian	1950
Ib4	Toba Batak	Austronesian	1930
Ib5	Kadazan-Dusun	Austronesian	1920
Ib6	Minangkabau	Austronesian	1920
Ic5	Toradja	Austronesian	1910
Ic6	Tanimbarese	Austronesian	1930
Ic8	Kei	Austronesian	1890
Ic9	Sumbanese	Austronesian	1930
Id1	Northern Aranda	Pama-Nyungan	1900
Id10	Walbiri	Pama-Nyungan	1930
Ie10	Motu	Austronesian	1950
Ie12	Kwoma	Sepik	1930
Ie17	Siane	Nuclear Trans New Guinea	1940
Ie2	Wantoat	Nuclear Trans New Guinea	1920
Ie20	Koita	Koiarian	1900
Ie25	Mafulu	Goilalan	1920
Ie38	Dani	Nuclear Trans New Guinea	1960
Ie4	Wogeo	Austronesian	1930
Ie5	Keraki	Morehead-Wasur	1930
Ie7	Enga	Nuclear Trans New Guinea	1950
Ie8	Purari	Purari	1910
If1	Palauans	Austronesian	1940
If14	Makin	Austronesian	1890
If2	Trukese	Austronesian	1940

If3	Majuro	Austronesian	1940
If5	Ponapeans	Austronesian	1910
If6	Yapese	Austronesian	1910
If7	Gilbert Onotoa	Austronesian	1940
If8	Chamorro	Austronesian	1950
Ig14	Dahuni	Austronesian	1900
Ig2	Trobriands	Austronesian	1910
Ig6	Ulawans	Austronesian	1900
Ig9	Manus	Austronesian	1920
Ij5	Raroians	Austronesian	1900
Ij7	Mangarevans	Austronesian	1900
Na1	Nabesna	Athapaskan-Eyak-Tlingit	1930
Na10	Chugach	Eskimo-Aleut	1930
Na12	Nunamiut	Eskimo-Aleut	1950
Na14	Inughuit (Northern Greenland)	Eskimo-Aleut	1880
Na2	Tareumiut	Eskimo-Aleut	1880
Na21	Caribou Inuit	Eskimo-Aleut	1900
Na22	Iglulik Inuit	Eskimo-Aleut	1920
Na23	Labrador Inuit	Eskimo-Aleut	1890
Na24	Tasiilaq	Eskimo-Aleut	1880
Na26	Dena'ina	Athapaskan-Eyak-Tlingit	1870
Na3	Copper Inuit	Eskimo-Aleut	1920
Na32	Montagnais	Algic	1880
Na37	Rainy River Ojibwe	Algic	1880
Na9	Aleut	Eskimo-Aleut	1830
Nb16	Klallam	Salishan	1860
Nb24	Makah	Wakashan	1860
Nb25	Quinault	Salishan	1860
Nb27	Stó:lō	Salishan	1880
Nb32	Shasta	Shastan	1860
Nb33	Chimariko	Chimariko	1860
Nb8	Haisla	Wakashan	1880
Nb9	Nuxalk	Salishan	1880
Nc11	Yana	Yana	1860
Nc12	Maidu	Maiduan	1850
Nc15	Coast Yuki	Yuki-Wappo	1860
Nc18	Eastern Pomo	Pomoan	1860
Nc19	Southern Pomo	Pomoan	1860
Nc2	Tübalulabal	Uto-Aztecan	1850
Nc21	Lake Miwok	Miwok-Costanoan	1860
Nc23	Western Mono	Uto-Aztecan	1870
Nc3	Northern Foothill Yokuts	Yokutsan	1850
Nc6	Kumeyaay	Cochimi-Yuman	1850
Nc7	Yuki	Yuki-Wappo	1850
Nc8	Klamath	Klamath-Modoc	1860
Nc9	Modoc	Klamath-Modoc	1860

Nd1	Tenino	Sahaptian	1850
Nd2	Southern Ute	Uto-Aztecan	1860
Nd20	Nez Perce	Sahaptian	1850
Nd24	Kidutokado	Uto-Aztecan	1870
Nd27	Kuyuidokado	Uto-Aztecan	1860
Nd3	Havasupai	Cochimi-Yuman	1870
Nd30	Eastern Mono	Uto-Aztecan	1870
Nd32	Tümpisa Shoshone (Saline and Panamint)	Uto-Aztecan	1850
Nd33	Beatty	Uto-Aztecan	1870
Nd43	White Knife Shoshoni	Uto-Aztecan	1860
Nd45	Bohogue	Uto-Aztecan	1860
Nd46	Agaiduka	Uto-Aztecan	1860
Nd47	Tukudika	Uto-Aztecan	1850
Nd52	Shivwits	Uto-Aztecan	1860
Nd55	Southern Paiute (Las Vegas)	Uto-Aztecan	1860
Nd57	Pahvant	Uto-Aztecan	1860
Nd6	Washo	Washo	1850
Nd63	Bannock	Uto-Aztecan	1860
Nd64	Wind River Eastern Shoshone	Uto-Aztecan	1860
Nd66	Yavapai	Cochimi-Yuman	1870
Nd67	Tolkepaya	Cochimi-Yuman	1870
Nd8	Chilcotin	Athapaskan-Eyak-Tlingit	1880
Nd9	Lillooet	Salishan	1860
Ne1	Gros Ventre	Algic	1880
Ne11	Assiniboine	Siouan	1870
Ne13	Kainai	Algic	1850
Ne18	Piegan	Algic	1850
Ne3	Comanche	Uto-Aztecan	1870
Ne4	Crow	Siouan	1870
Ne5	Cheyenne	Algic	1860
Ne9	Arapaho	Algic	1860
Nf13	Shawnee	Algic	1820
Nf3	Omaha	Siouan	1850
Nf6	Pawnee	Caddoan	1867
Nh1	Chiricahua	Athapaskan-Eyak-Tlingit	1880
Nh10	Isleta	Kiowa-Tanoan	1920
Nh11	San Ildefonso	Kiowa-Tanoan	1900
Nh12	Santa Ana	Keresan	1920
Nh16	Jicarilla	Athapaskan-Eyak-Tlingit	1870
Nh17	Western Apache	Athapaskan-Eyak-Tlingit	1870
Nh20	Kamia	Cochimi-Yuman	1860
Nh25	Sia	Keresan	1920
Ni1	Tarahumara	Uto-Aztecan	1930

Ni2	Tohono O'odham	Uto-Aztecan	1930
Ni6	Pima	Uto-Aztecan	1840
Nj9	Tlaxcalans	Uto-Aztecan	1960
Sa8	Mam	Mayan	1930
Sb2	Cágaba	Chibchan	1940
Sb4	Cariña	Cariban	1950
Sc18	Ndyuka	Indo-European	1960
Sc2	Pumé	Pumé	1950
Sc4	Guahibo	Guahibo	1950
Sd1	Munduruku	Tupian	1950
Se1	Sirionó	Tupian	1940
Se3	Shuar	Jivaroan	1930
Se4	Yagua	Peba-Yagua	1940
Se5	Cubeo	Tucanoan	1940
Sg1	Yahgan	Yámana	1870
Sg3	Ona	Chonan	1880
Sh8	Toba	Guaicuruan	1964
Si5	Kamayurá	Tupian	1940
Sj3	Aweikoma	Nuclear-Macro-Je	1910
Sj6	Tenetehara	Tupian	1930

Distribution of variables across large and small scale societies

Because subsistence and inequality have been hypothesized to be associated with political complexity, we examined the distribution of levels of political hierarchy (coded in variable *EA033: Jurisdictional hierarchy beyond local community*) for the 164 societies in our dataset that practice intensive agriculture and for which EA033 data was available, and for the 138 societies in our dataset with heritable social class for which EA033 data was available (*12*). Inspection of these distributions shows that intensive agriculture is not confined or skewed toward large scale societies, and that the inequality phenomena we target are found in a range of societies, and not only in highly politically complex societies.

Fig. S1. Distribution of Intensive Agriculture and inequality variables across levels of Political Complexity.

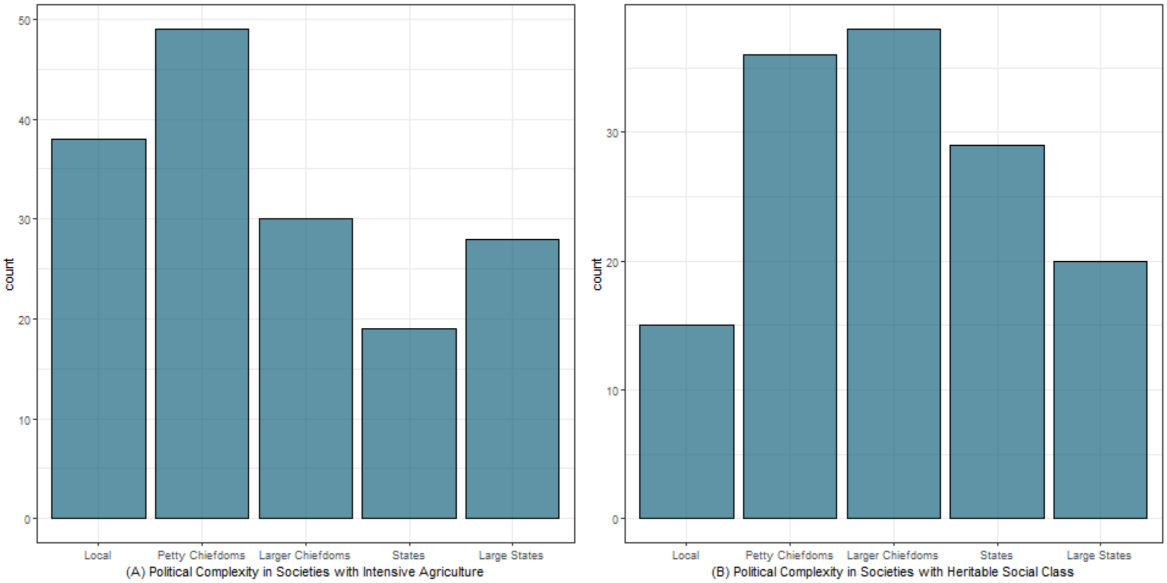

Number of societies coded (1: present) for (**A**) Intensive Agriculture, and (**B**) Heritable Social Class variables across levels of political hierarchy. X-axis represents political complexity categories encoded in *EA033*. Y-axis represents number of societies coded (1) for the binary response variable of interest.

Principal Component Analysis of environmental data

We used a principal component analysis to derive a more independent set of variables from a larger suite of environmental data that includes known multicollinearities.

Table S2: Results of Principal Component Analysis of Environmental Variables.

Variable	PC1	PC2	PC3	Uniqueness
Temperature predictability	**0.93**	-0.02	-0.21	0.082
ln (Annual temperature variance)	**-0.92**	0.04	0.22	0.105
NPP predictability	**-0.84**	-0.39	0.00	0.151
Annual mean temperature	**0.81**	-0.33	-0.31	0.146
sqrt (Annual mean precipitation)	**0.71**	0.54	0.03	0.201
ln (Annual precipitation variance)	**0.69**	0.29	0.21	0.392
sqrt (Annual mean NPP)	**0.64**	0.58	0.09	0.244
ln (Annual mean NPP variance)	-0.10	**0.85**	0.04	0.269
Precipitation predictability	0.27	**0.73**	-0.20	0.351
ln (Slope)	-0.09	0.07	**0.89**	0.185
ln (Elevation)	-0.06	-0.15	**0.88**	0.202
SS loadings	4.57	2.25	1.86	
Cumulative variance	0.42	0.62	0.79	

Expanded results from stepwise model

Table S3. Tests of directed separation from piecewise SEM for stepwise model (Global goodness-of-fit: Fisher's C = 65.598 with P-value = 0 and on 28 degrees of freedom)**.**

Independence Claim	Critical Value	P-Value
Inheritance_Real_Unigeniture ~ PC1 + ...	0.1330	0.8942
Inheritance_Move_ Unigeniture ~ PC1 + ...	0.9916	0.3214
Succession ~ PC1 + ...	2.9153	0.0036
Class ~ PC1 + ...	-0.9866	0.3238
Inheritance_Real_ Unigeniture ~ PC2 + ...	1.5461	0.1221
Inheritance_Move_ Unigeniture ~ PC2 + ...	-0.2534	0.8000
Succession ~ PC2 + ...	-1.5878	0.1123
Class ~ PC2 + ...	-0.0399	0.9682
Inheritance_Real_ Unigeniture ~ PC3 + ...	-0.9458	0.3443
Inheritance_Move_ Unigeniture ~ PC3 + ...	0.9623	0.3359
Succession ~ PC3 + ...	0.1529	0.8785
Class ~ PC3 + ...	-0.1602	0.8728
Class ~ IntensiveAg_Binary + ...	4.6804	0.0000
Class ~ Large_Animals + ...	2.7272	0.0064

Table S4. Estimates for paths in stepwise model

Response	Predictor	Estimate	Std Error	Critical Value	P-Value
Intensive Ag	PC1	0.1123	0.2777	0.4043	0.6860
Intensive Ag	PC2	-0.6311	0.2195	-2.8753	0.0040
Intensive Ag	PC3	0.3233	0.1657	1.9511	0.0510
Large_Animals	PC1	-1.1566	0.3252	-3.5568	0.0004
Large_Animals	PC2	-0.8344	0.2104	-3.9660	0.0001
Large_Animals	PC3	0.4255	0.1770	2.4042	0.0162
Large_Animals	IntensiveAg_Binary	1.1347	0.3355	3.3820	0.0007
Inheritance_Real_Unigen	IntensiveAg_Binary	0.0127	0.2919	0.0437	0.9652
Inheritance_Real_Unigen	Large_Animals	0.1785	0.2836	0.6293	0.5292
Inheritance_Move_Unigen	IntensiveAg	-0.2662	0.4603	-0.5784	0.5630
Inheritance_Move_Unigen	Large_Animals	0.6466	0.4205	1.5379	0.1241
Inheritance_Move_Unigen	Inheritance_Real_Unigen	4.5794	0.4614	9.9255	0.0000
Succession	IntensiveAg	-0.3008	0.2501	-1.2030	0.2290
Succession	Large_Animals	-0.8706	0.2499	-3.4835	0.0005
Succession	Inheritance_Real_Unigen	0.3688	0.3591	1.0270	0.3044

Succession	Inheritance_Move_Unigen	0.7112	0.3780	1.8815	0.0599
Class	Inheritance_Real_Unigen	0.8259	0.4081	2.0238	0.0430
Class	Inheritance_Move_Unigen	-0.2641	0.4331	-0.6099	0.5419
Class	Succession	0.5349	0.2617	2.0444	0.0409

Table S5. Marginal and Conditional R^2 values for all response variables in stepwise model.

Response Variable	Marginal	Conditional
IntensiveAg	0.05	0.63
Large_Animals	0.21	0.68
Inheritance_Real_Unigeniture	0.00	0.25
Inheritance_Move_ Unigeniture	0.40	0.55
Succession	0.10	0.16
Class	0.03	0.30

Expanded results from full model

Table S6. Estimates (standardized) for paths in full model.

Response	Predictor	Std. Estimate	Std. Error	Crit. Value	p
Intensive Agriculture	Environmental Productivity (PC1)	0.112	0.278	0.404	0.686
Intensive Agriculture	Environmental Variability (PC2)	-0.631	0.220	-2.875	0.004
Intensive Agriculture	Slope and Elevation (PC3)	0.323	0.166	1.951	0.051
Large Domestic Animals	Environmental Productivity (PC1)	-1.157	0.325	-3.557	0.000
Large Domestic Animals	Environmental Variability (PC2)	-0.834	0.210	-3.966	0.000
Large Domestic Animals	Slope and Elevation (PC3)	0.426	0.177	2.404	0.016
Large Domestic Animals	Intensive Agriculture	1.135	0.336	3.382	0.001
Population Size	Environmental Productivity (PC1)	-0.090	0.086	1.096	0.296
Population Size	Environmental Variability (PC2)	0.082	0.061	1.772	0.184
Population Size	Slope and Elevation (PC3)	0.023	0.051	0.207	0.650
Population Size	Intensive Agriculture	0.078	0.109	2.097	0.148
Population Size	Large Domestic Animals	0.049	0.106	0.871	0.351
Real Property Unigeniture	Intensive Agriculture	-0.004	0.292	-0.015	0.988
Real Property Unigeniture	Large Domestic Animals	0.174	0.283	0.614	0.540
Real Property Unigeniture	Population Size	0.101	0.141	0.719	0.472
Movable Property Unigeniture	Intensive Agriculture	-0.285	0.462	-0.616	0.538
Movable Property Unigeniture	Large Domestic Animals	0.638	0.421	1.516	0.130
Movable Property Unigeniture	Population Size	0.083	0.228	0.364	0.716
Movable Property Unigeniture	Real Property Unigeniture	4.573	0.461	9.917	0.000
Hereditary Political Succession	Intensive Agriculture	-0.268	0.252	-1.063	0.288
Hereditary Political Succession	Large Domestic Animals	-0.848	0.250	-3.389	0.001
Hereditary Political Succession	Population Size	-0.247	0.317	-0.781	0.435
Hereditary Political Succession	Real Property Unigeniture	0.372	0.359	1.039	0.299
Hereditary Political Succession	Movable Property Unigeniture	0.718	0.378	1.899	0.058

Heritable Social Class	Population Size	1.495	0.697	2.144	0.032
Heritable Social Class	Real Property Unigeniture	0.918	0.426	2.156	0.031
Heritable Social Class	Movable Property Unigeniture	-0.373	0.453	-0.824	0.410
Heritable Social Class	Large Domestic Animals	0.653	0.289	2.261	0.024
Heritable Social Class	Intensive Agriculture	1.032	0.300	3.441	0.001
Heritable Social Class	Hereditary Political Succession	0.848	0.278	3.046	0.002

Table S7. Marginal and Conditional R^2 values for all response variables in full model.

Variable	Marginal R2	Conditional R2
Intensive Agriculture	0.05	0.63
Large Domesticated Animals	0.21	0.68
Population Size	0.01	0.58
Real Property Unigeniture	0.00	0.25
Movable Property Unigeniture	0.40	0.55
Hereditary Political Succession	0.11	0.17
Heritable Social Class	0.31	0.45

Table S8. Tests of directed separation from piecewise SEM for full model (Fisher's C = 32.597 with P-value = 0.113 and on 24 degrees of freedom).

Independence Claim	Crit.Value	P-Value
Inheritance_Real_Unigen ~ PC1 + ...	0.1805	0.8568
Inheritance_Move_Unigen ~ PC1 + ...	0.9968	0.3189
Succession ~ PC1 + ...	2.9250	0.0034
Class ~ PC1 + ...	-0.5760	0.5646
Inheritance_Real_Unigen ~ PC2 + ...	1.5047	0.1324
Inheritance_Move_Unigen ~ PC2 + ...	-0.2946	0.7683
Succession ~ PC2 + ...	-1.5302	0.1260
Class ~ PC2 + ...	1.2726	0.2031
Inheritance_Real_Unigen ~ PC3 + ...	-0.9599	0.3371
Inheritance_Move_Unigen ~ PC3 + ...	0.9542	0.3400
Succession ~ PC3 + ...	0.1264	0.8994
Class ~ PC3 + ...	-0.5452	0.5856

Results with pastoralism-dominant societies excluded

To examine the possibility that both the nature of large animal resources and their use in pastoral societies, as well as the political, economic, and social characteristics of pastoral societies differ from agriculturalists in ways that might obscure the role of large domesticated animals in this network of effects, we ran the analyses again after excluding from the sample all societies whose dominant mode of subsistence is pastoralism.

To do this, we examined the values for the variable *EA042: Subsistence economy dominant activity* (*12*), and removed all societies from the sample that were coded 4 (*Pastoralism*

contributes most). The resulting sample contained 387 societies. Principal components were recalculated with this sample before proceeding with analyses, and both components and model results were found to be qualitatively similar to the analyses reported in the main text. Excluded societies are Aa3 *Nama*, Ab1 *Herero*, Aj2 *Maasi*, Aj23 *Plains Suk*, Aj28 *Tatoga,* Aj29 *Samburu*, Aj3 *Nuer*, Ca10 *Esa*, Ca38 *Tigre*, Cb8 *Bororo Fulani*, Cc1 *Regeibat*, Cc10 *Asben*, Cc11 *Azjer*, Cd18 *Saadi*, Ci1 *Kalmyk*, Ci6 *Ossetians*, Ea10 *Marri*, Ec2 *Yakut*, Ec3 *Chukchi*, Eg4 *Toda*, and Eh7 *Antandroy*.

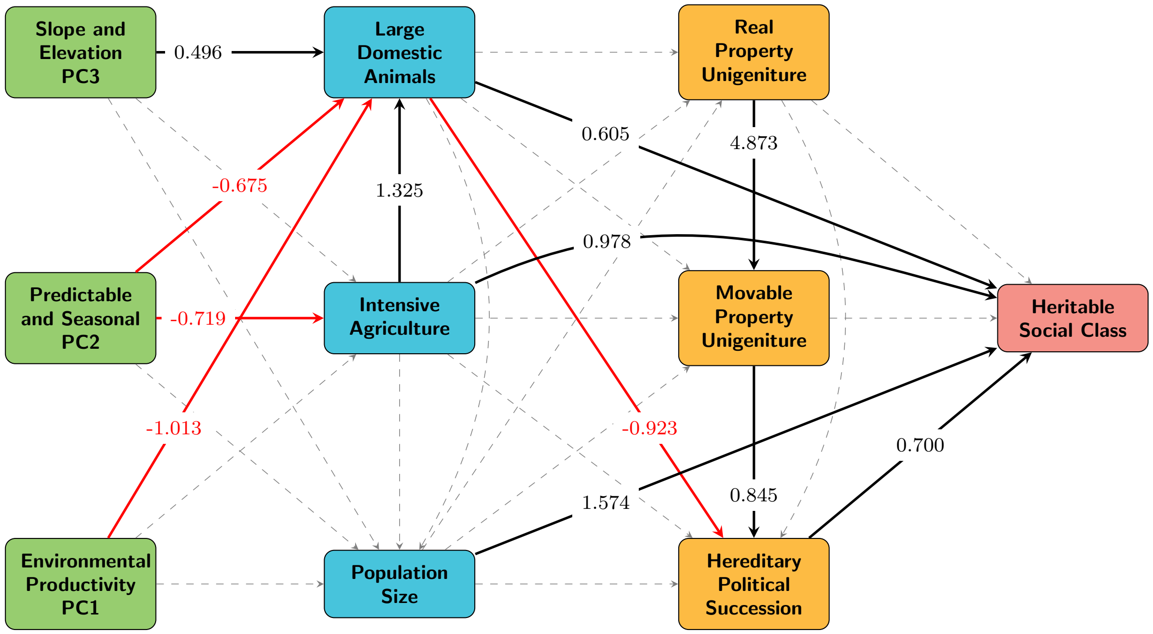

Fig. S2. Path diagram representing full model, excluding societies whose dominant subsistence strategy is pastoralism. Red arrows indicate negative relationships identified in PiecewiseSEM model. Black arrows represent positive relationships identified in PiecewiseSEM model. Dashed arrows represent paths not found to be significant ($p < 0.05$). Significant paths are labeled with standardized coefficients. Individual variables represented by boxes in the diagram can be interpreted as *increasing* for continuous variables and *present* for categorical variables. Fisher's C = 34.964, p = 0.069. Heritable Social Class Marginal R^2 = 0.33, Conditional R^2 = 0.45. n=387.

Results with alternative coding of inheritance

All analyses were run with an alternative coding of Real Property Inheritance and Movable Property Inheritance to examine variation in results when the inheritance patterns considered do not inherently concentrate wealth among a subset of potential heirs. In this model intergenerational wealth transfer variables are coded as follows:

- Real Property Inheritance – a binary variable expressing the presence or absence of any norm for inheritance of land, recoded from *EA074 Inheritance rule for real property* (*12*).

– 0: EA074 = 1 *No inheritance of real property*

– 1: EA074 = 2 *Matrilineal by sister's sons* OR 3 *Matrilineal by heirs* OR 4 *Children, less for daughters* OR 5 *Children* OR 6 *Patrilineal by heirs* OR 7 *Patrilineal by sons*

- Movable Property Inheritance – a binary variable expressing the presence or absence of any norm for inheritance of movable property and wealth, recoded from EA076 *Inheritance rule for moveable property* (*12*).

– 0: EA076 = 1 *No inheritance of movable property*

– 1: EA076 = 2 *Matrilineal by sister's sons* OR 3 *Matrilineal by heirs* OR 4 *Children, less for daughters* OR 5 *Children* OR 6 *Patrilineal by heirs* OR 7 *Patrilineal by sons*

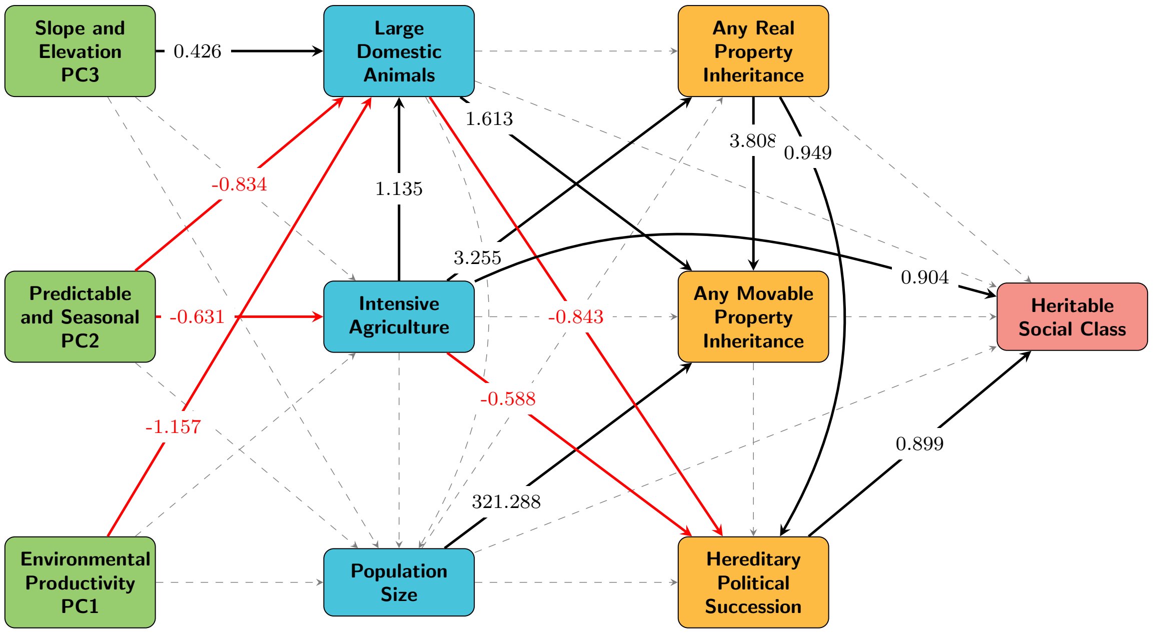

Fig. S3: Path diagram with alternative characterization of inheritance. Path diagram representing full model, with transmission of wealth coded as presence or absence of any inheritance rules. Red arrows indicate negative relationships identified in PiecewiseSEM model. Black arrows represent positive relationships identified in PiecewiseSEM model. Dashed arrows represent paths not found to be significant (p <0.05). Significant paths are labeled with standardized coefficients. Individual variables represented by boxes in the diagram can be interpreted as *increasing* for continuous variables and *present* for categorical variables. Fisher's C = 52.785, p = 0.001. Heritable Social Class Marginal R^2 = 0.31, Conditional R^2 = 0.43. n=408.
